# Poly[diaqua­bis(μ^2^-azido-κ^2^ *N* ^1^:*N* ^1^)bis­(μ_3_-1-oxoisonicotinato-κ^3^ *O*:*O*′:*O*′′)dicadmium(II)]

**DOI:** 10.1107/S1600536808013196

**Published:** 2008-05-10

**Authors:** Zhi-Xiang Wang, Xiu-Bing Li, Bai-Wang Sun

**Affiliations:** a Ordered Matter Science Research Center, College of Chemistry and Chemical Engineering, Southeast University, Nanjing 210096, People’s Republic of China; b Department of Chemistry, Key Laboratory of Medicinal Chemistry for Natural Resources, Ministry of Education, Yunnan University, Kunming 650091, People’s Republic of China

## Abstract

In the title compound, [Cd_2_(C_6_H_4_NO_3_)_2_(N_3_)_2_(H_2_O)_2_]_*n*_, one Cd^II^ atom is located on an inversion center and is coordinated by four O atoms from four bridging 1-oxoisonicotinate ligands and two N atoms of two bridging azide ligands in a slightly distorted octa­hedral geometry. The other Cd^II^ atom, also lying on an inversion center, is coordinated by four O atoms from two bridging 1-oxoisonicotinate ligands and two water mol­ecules and two N atoms of two bridging azide ligands in a slightly distorted octa­hedral geometry. The Cd atoms are connected *via* the 1-oxoisonicotinate and azide ligands into a two-dimensional coordination network. The crystal structure involves O—H⋯N and O—H⋯O hydrogen bonds.

## Related literature

For general background, see: Du *et al.* (2006 ▶); Dybtsev *et al.* (2004 ▶). For related structures, see: Bai *et al.* (2004 ▶); He *et al.* (2005 ▶); Zhao *et al.* (2007 ▶).
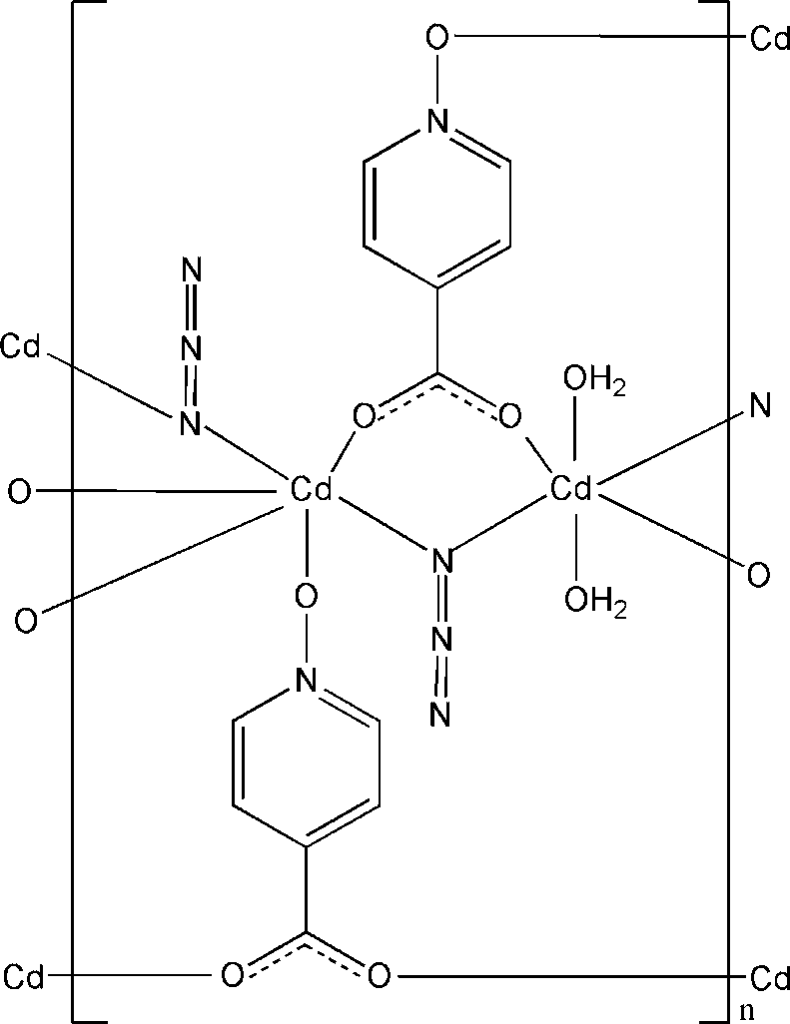


## Experimental

### 

#### Crystal data

[Cd_2_(C_6_H_4_NO_3_)_2_(N_3_)_2_(H_2_O)_2_]
*M* *_r_* = 621.10
Triclinic, 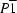

*a* = 6.5409 (17) Å
*b* = 7.850 (2) Å
*c* = 9.410 (3) Å
α = 99.668 (6)°
β = 97.164 (6)°
γ = 107.566 (5)°
*V* = 446.1 (2) Å^3^
*Z* = 1
Mo *K*α radiation
μ = 2.45 mm^−1^
*T* = 223 (2) K
0.3 × 0.2 × 0.2 mm

#### Data collection

Rigaku Scxmini 1K CCD area-detector diffractometer
Absorption correction: multi-scan (*CrystalClear*; Rigaku, 2005 ▶) *T*_min_ = 0.612, *T*_max_ = 0.613
5082 measured reflections
1567 independent reflections
1438 reflections with *I* > 2σ(*I*)
*R*_int_ = 0.022

#### Refinement

*R*[*F* ^2^ > 2σ(*F* ^2^)] = 0.033
*wR*(*F* ^2^) = 0.087
*S* = 1.08
1567 reflections
139 parameters
1 restraint
H-atom parameters constrained
Δρ_max_ = 0.60 e Å^−3^
Δρ_min_ = −1.02 e Å^−3^

### 

Data collection: *CrystalClear* (Rigaku, 2005 ▶); cell refinement: *CrystalClear*; data reduction: *CrystalClear*; program(s) used to solve structure: *SHELXS97* (Sheldrick, 2008 ▶); program(s) used to refine structure: *SHELXL97* (Sheldrick, 2008 ▶); molecular graphics: *Mercury* (Macrae *et al.*, 2006 ▶) and *SHELXTL* (Sheldrick, 2008 ▶); software used to prepare material for publication: *SHELXTL*.

## Supplementary Material

Crystal structure: contains datablocks I, global. DOI: 10.1107/S1600536808013196/hy2123sup1.cif

Structure factors: contains datablocks I. DOI: 10.1107/S1600536808013196/hy2123Isup2.hkl

Additional supplementary materials:  crystallographic information; 3D view; checkCIF report

## Figures and Tables

**Table:** 

Cd1—N1	2.259 (3)
Cd1—O1	2.289 (3)
Cd1—O3^i^	2.370 (3)
Cd2—O2	2.242 (3)
Cd2—N1^ii^	2.284 (3)
Cd2—O4	2.363 (3)

**Table:** 

N1—Cd1—O1	85.90 (12)
N1^iii^—Cd1—O1	94.10 (12)
N1—Cd1—O3^i^	90.18 (12)
O1—Cd1—O3^i^	89.01 (11)
N1—Cd1—O3^iv^	89.82 (12)
O2—Cd2—N1^ii^	85.06 (12)
O2—Cd2—O4^v^	87.87 (12)
O2^v^—Cd2—O4^v^	92.13 (12)
N1^ii^—Cd2—O4^v^	94.08 (12)
N1^iii^—Cd2—O4^v^	85.92 (12)

Symmetry codes: (i) 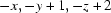; (ii) 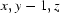; (iii) 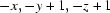; (iv) 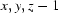; (v) 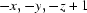.

**Table 2:** Hydrogen-bond geometry (Å, °)

*D*—H⋯*A*	*D*—H	H⋯*A*	*D*⋯*A*	*D*—H⋯*A*
O4—H4*C*⋯N3^vi^	0.90	2.36	3.239 (7)	167
O4—H4*B*⋯O3^vii^	0.83	2.05	2.716 (4)	137

Symmetry codes: (vi) 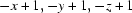; (vii) 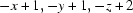.

**Fig. 1.**
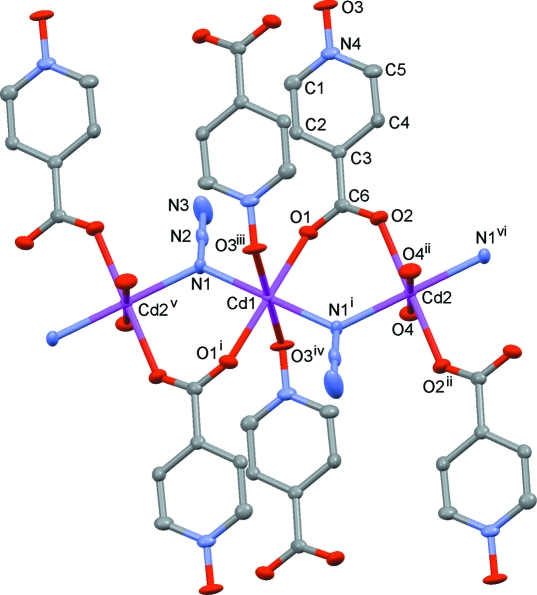
The asymmetric unit of the title compound, together with symmetry-related atoms to complete the coordination units. Displacement ellipsoids are drawn at the 30% probability level. H atoms have been omitted for clarity. [Symmetry codes: (i) -x, -y + 1, -z + 1; (ii) -x, -y, 1 - z; (iii) -x, 1 - y, 2 - z; (iv) x, y, -1 + z; (v) x, 1 + y, z; (vi) x, -1 + y, z.]

**Fig. 2.**
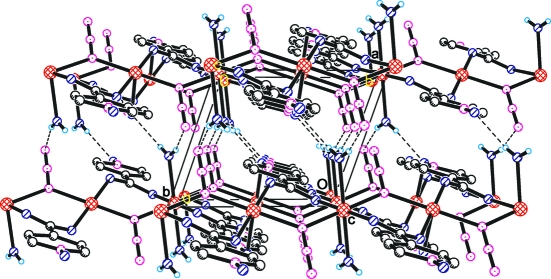
Crystal packing of the title compound. Hydrogen bonds are shown as dashed lines.

## supplementary crystallographic information

### Comment

There is currently considerable interest in the synthesis and characterization of metal–organic frameworks because of their potential applications in molecular adsorption and separation processes, gas storage, ion exchange, catalysis, sensor technology and electronics (Du *et al.*, 2006; Dybtsev *et al.*, 2004). The isonicotinic acid N-oxide ligand possesses a longer bridging spacer and richer coordination modes to form a fascinating structure (He *et al.*, 2005; Zhao *et al.*, 2007). It is well known that azide anion is an excellent bridging ligand (Bai *et al.*, 2004). Therefore, we expect to obtain higher dimensional structures based on isonicotinic acid N-oxide and azide ligands and transition metal ions through the control of their molar ratios. We report here the synthesis and crystal structure of the title compound.

In the title compound, the Cd1 atom is located on an inversion center and is coordinated by four O atoms from four bridging isonicotinate-N-oxide ligands and two N atoms of two bridging azide ligands in a slightly distorted octahedral geometry. The Cd2 atom, also lying on an inversion center, is coordinated by four O atoms from two bridging isonicotinate-N-oxide ligands and two water molecules and two N atoms of two azide ligands in a slightly distorted octahedral geometry (Fig. 1; Table 1). The Cd atoms are connected *via* the isonicotinate-N-oxide and azide ligands into a two-dimensional coordination network. Furthermore, a three-dimensional supramolecular network is formed by the intermolecular O—H···N and O—H···O hydrogen bonds (Fig. 2; Table 2).

### Experimental

All reagents and solvents were used as obtained without further purification. Cd(NO_3_)_2_.4H_2_O (0.062 g, 0.2 mmol), isonicotinic acid N-oxide (0.028 g, 0.2 mmol), NaN_3_ (0.013 g, 0.2 mmol) and NaOH (0.016 g, 0.4 mmol) were dissolved in distilled water (10 ml). The mixture was sealed in a Teflon-lined stainless steel vessel and held at 443 K for one week. The vessel was gradually cooled to room temperature and colorless crystals suitable for crystallographic analysis were obtained.

### Refinement

H atoms on C atoms were positioned geometrically and refined as riding atoms, with C–H = 0.93 Å and *U*_iso_(H) = 1.2*U*_eq_(C). H atoms of the water molecule were located in a difference Fourier map and fixed in the refinements with *U*_iso_(H) = 1.2*U*_eq_(O).

### Crystal data

**Table:**

[Cd_2_(C_6_H_4_NO_3_)_2_(N_3_)_2_(H_2_O)_2_]	*Z* = 1
*M**_r_* = 621.10	*F*(000) = 300
Triclinic, *P*1	*D*_x_ = 2.312 Mg m^−^^3^
Hall symbol: -P 1	Mo *K*α radiation, λ = 0.71073 Å
*a* = 6.5409 (17) Å	Cell parameters from 2159 reflections
*b* = 7.850 (2) Å	θ = 3.1–26.8°
*c* = 9.410 (3) Å	µ = 2.45 mm^−^^1^
α = 99.668 (6)°	*T* = 223 K
β = 97.164 (6)°	Block, colorless
γ = 107.566 (5)°	0.3 × 0.2 × 0.2 mm
*V* = 446.1 (2) Å^3^	

### Data collection

**Table:**

Rigaku Scxmini 1K CCD area-detector diffractometer	1567 independent reflections
Radiation source: fine-focus sealed tube	1438 reflections with *I* > 2σ(*I*)
graphite	*R*_int_ = 0.022
Detector resolution: 8.192 pixels mm^-1^	θ_max_ = 25.0°, θ_min_ = 2.8°
thin–slice ω scans	*h* = −7→7
Absorption correction: multi-scan (*CrystalClear*; Rigaku, 2005)	*k* = −9→7
*T*_min_ = 0.612, *T*_max_ = 0.613	*l* = −11→10
5082 measured reflections	

### Refinement

**Table:**

Refinement on *F*^2^	Primary atom site location: structure-invariant direct methods
Least-squares matrix: full	Secondary atom site location: difference Fourier map
*R*[*F*^2^ > 2σ(*F*^2^)] = 0.033	Hydrogen site location: inferred from neighbouring sites
*wR*(*F*^2^) = 0.087	H-atom parameters constrained
*S* = 1.08	*w* = 1/[σ^2^(*F*_o_^2^) + (0.05*P*)^2^ + 1.3747*P*] where *P* = (*F*_o_^2^ + 2*F*_c_^2^)/3
1567 reflections	(Δ/σ)_max_ < 0.001
139 parameters	Δρ_max_ = 0.60 e Å^−^^3^
1 restraint	Δρ_min_ = −1.02 e Å^−^^3^

### Fractional atomic coordinates and isotropic or equivalent isotropic displacement parameters (Å^2^)

**Table:**

	*x*	*y*	*z*	*U*_iso_*/*U*_eq_	
Cd1	0.0000	0.5000	0.5000	0.01556 (17)	
Cd2	0.0000	0.0000	0.5000	0.01683 (17)	
O1	0.1884 (5)	0.4600 (4)	0.7066 (3)	0.0244 (7)	
O2	0.0598 (6)	0.1674 (4)	0.7283 (3)	0.0311 (8)	
O3	0.3130 (5)	0.5495 (5)	1.3892 (3)	0.0270 (7)	
O4	0.3676 (5)	0.1301 (5)	0.4778 (4)	0.0295 (8)	
H4B	0.4361	0.2055	0.5546	0.035*	
H4C	0.4127	0.0951	0.3945	0.035*	
N1	0.1164 (6)	0.7997 (5)	0.6110 (4)	0.0201 (8)	
N2	0.2664 (6)	0.8640 (5)	0.7131 (4)	0.0227 (8)	
N3	0.4108 (8)	0.9283 (6)	0.8078 (6)	0.0506 (14)	
N4	0.2847 (6)	0.5005 (5)	1.2437 (4)	0.0208 (8)	
C1	0.3105 (6)	0.6288 (6)	1.1625 (5)	0.0213 (9)	
H1A	0.3534	0.7529	1.2095	0.026*	
C2	0.2752 (6)	0.5808 (6)	1.0126 (5)	0.0171 (8)	
H2A	0.2954	0.6714	0.9569	0.021*	
C3	0.2086 (6)	0.3959 (6)	0.9430 (4)	0.0168 (8)	
C4	0.1925 (7)	0.2671 (6)	1.0312 (5)	0.0216 (9)	
H4A	0.1547	0.1423	0.9873	0.026*	
C5	0.2314 (7)	0.3216 (6)	1.1804 (5)	0.0240 (9)	
H5A	0.2211	0.2345	1.2391	0.029*	
C6	0.1483 (7)	0.3368 (6)	0.7782 (5)	0.0189 (9)	

### Atomic displacement parameters (Å^2^)

**Table:**

	*U*^11^	*U*^22^	*U*^33^	*U*^12^	*U*^13^	*U*^23^
Cd1	0.0215 (3)	0.0136 (2)	0.0112 (2)	0.00553 (18)	0.00313 (17)	0.00218 (17)
Cd2	0.0216 (3)	0.0152 (3)	0.0132 (3)	0.00717 (18)	0.00221 (17)	0.00086 (17)
O1	0.0288 (16)	0.0273 (17)	0.0148 (15)	0.0069 (14)	0.0003 (12)	0.0050 (13)
O2	0.049 (2)	0.0251 (18)	0.0161 (15)	0.0136 (16)	0.0021 (14)	−0.0019 (13)
O3	0.0253 (16)	0.0362 (19)	0.0095 (15)	−0.0004 (14)	0.0045 (12)	−0.0036 (13)
O4	0.0259 (17)	0.0337 (19)	0.0214 (17)	0.0014 (15)	0.0050 (14)	0.0010 (14)
N1	0.028 (2)	0.0120 (16)	0.0175 (18)	0.0069 (15)	−0.0020 (16)	0.0005 (14)
N2	0.027 (2)	0.0149 (17)	0.027 (2)	0.0079 (16)	0.0011 (19)	0.0071 (16)
N3	0.054 (3)	0.024 (2)	0.055 (3)	0.004 (2)	−0.029 (3)	0.003 (2)
N4	0.0178 (17)	0.028 (2)	0.0146 (17)	0.0054 (15)	0.0059 (14)	0.0020 (15)
C1	0.0146 (19)	0.024 (2)	0.022 (2)	0.0038 (17)	0.0020 (17)	0.0015 (18)
C2	0.0159 (19)	0.020 (2)	0.018 (2)	0.0079 (16)	0.0050 (16)	0.0061 (16)
C3	0.0133 (18)	0.020 (2)	0.017 (2)	0.0061 (16)	0.0044 (16)	0.0029 (16)
C4	0.030 (2)	0.017 (2)	0.020 (2)	0.0099 (18)	0.0079 (18)	0.0047 (17)
C5	0.025 (2)	0.028 (2)	0.017 (2)	0.0057 (19)	0.0076 (18)	0.0046 (18)
C6	0.017 (2)	0.027 (2)	0.016 (2)	0.0120 (18)	0.0034 (16)	0.0047 (18)

### Geometric parameters (Å, °)

**Table:**

Cd1—N1	2.259 (3)	O4—H4B	0.8300
Cd1—N1^i^	2.259 (3)	O4—H4C	0.9000
Cd1—O1	2.289 (3)	N1—N2	1.201 (5)
Cd1—O1^i^	2.289 (3)	N1—Cd2^vii^	2.284 (3)
Cd1—O3^ii^	2.370 (3)	N2—N3	1.137 (6)
Cd1—O3^iii^	2.370 (3)	N4—C1	1.347 (6)
Cd2—O2	2.242 (3)	N4—C5	1.349 (6)
Cd2—O2^iv^	2.242 (3)	C1—C2	1.369 (6)
Cd2—N1^v^	2.284 (3)	C1—H1A	0.9400
Cd2—N1^i^	2.284 (3)	C2—C3	1.397 (6)
Cd2—O4^iv^	2.363 (3)	C2—H2A	0.9400
Cd2—O4	2.363 (3)	C3—C4	1.401 (6)
O1—C6	1.252 (5)	C3—C6	1.507 (6)
O2—C6	1.257 (5)	C4—C5	1.365 (6)
O3—N4	1.332 (5)	C4—H4A	0.9400
O3—Cd1^vi^	2.370 (3)	C5—H5A	0.9400
			
N1—Cd1—N1^i^	180.000 (1)	C6—O2—Cd2	130.6 (3)
N1—Cd1—O1	85.90 (12)	N4—O3—Cd1^vi^	118.5 (2)
N1^i^—Cd1—O1	94.10 (12)	Cd2—O4—H4B	109.5
N1—Cd1—O1^i^	94.10 (12)	Cd2—O4—H4C	120.1
N1^i^—Cd1—O1^i^	85.90 (12)	H4B—O4—H4C	130.4
O1—Cd1—O1^i^	180.0	N2—N1—Cd1	122.0 (3)
N1—Cd1—O3^ii^	90.18 (12)	N2—N1—Cd2^vii^	117.2 (3)
N1^i^—Cd1—O3^ii^	89.82 (12)	Cd1—N1—Cd2^vii^	119.53 (15)
O1—Cd1—O3^ii^	89.01 (11)	N3—N2—N1	178.2 (5)
O1^i^—Cd1—O3^ii^	90.99 (11)	O3—N4—C1	120.0 (4)
N1—Cd1—O3^iii^	89.82 (12)	O3—N4—C5	118.9 (4)
N1^i^—Cd1—O3^iii^	90.18 (12)	C1—N4—C5	121.2 (4)
O1—Cd1—O3^iii^	90.99 (11)	N4—C1—C2	120.9 (4)
O1^i^—Cd1—O3^iii^	89.01 (11)	N4—C1—H1A	119.5
O3^ii^—Cd1—O3^iii^	180.0	C2—C1—H1A	119.5
O2—Cd2—O2^iv^	180.0	C1—C2—C3	119.4 (4)
O2—Cd2—N1^v^	85.06 (12)	C1—C2—H2A	120.3
O2^iv^—Cd2—N1^v^	94.94 (12)	C3—C2—H2A	120.3
O2—Cd2—N1^i^	94.94 (12)	C2—C3—C4	118.0 (4)
O2^iv^—Cd2—N1^i^	85.06 (12)	C2—C3—C6	120.9 (4)
N1^v^—Cd2—N1^i^	180.000 (1)	C4—C3—C6	121.1 (4)
O2—Cd2—O4^iv^	87.87 (12)	C5—C4—C3	120.4 (4)
O2^iv^—Cd2—O4^iv^	92.13 (12)	C5—C4—H4A	119.8
N1^v^—Cd2—O4^iv^	94.08 (12)	C3—C4—H4A	119.8
N1^i^—Cd2—O4^iv^	85.92 (12)	N4—C5—C4	119.9 (4)
O2—Cd2—O4	92.13 (12)	N4—C5—H5A	120.0
O2^iv^—Cd2—O4	87.87 (12)	C4—C5—H5A	120.0
N1^v^—Cd2—O4	85.92 (12)	O1—C6—O2	127.4 (4)
N1^i^—Cd2—O4	94.08 (12)	O1—C6—C3	117.1 (4)
O4^iv^—Cd2—O4	180.0	O2—C6—C3	115.5 (4)
C6—O1—Cd1	132.5 (3)		
			
N1—Cd1—O1—C6	141.5 (4)	O3—N4—C1—C2	−177.8 (4)
N1^i^—Cd1—O1—C6	−38.5 (4)	C5—N4—C1—C2	2.5 (6)
O3^ii^—Cd1—O1—C6	51.2 (4)	N4—C1—C2—C3	0.8 (6)
O3^iii^—Cd1—O1—C6	−128.8 (4)	C1—C2—C3—C4	−3.5 (6)
N1^v^—Cd2—O2—C6	136.6 (4)	C1—C2—C3—C6	174.4 (4)
N1^i^—Cd2—O2—C6	−43.4 (4)	C2—C3—C4—C5	2.9 (6)
O4^iv^—Cd2—O2—C6	−129.1 (4)	C6—C3—C4—C5	−174.9 (4)
O4—Cd2—O2—C6	50.9 (4)	O3—N4—C5—C4	177.2 (4)
O1—Cd1—N1—N2	17.4 (4)	C1—N4—C5—C4	−3.1 (6)
O1^i^—Cd1—N1—N2	−162.6 (4)	C3—C4—C5—N4	0.3 (7)
O3^ii^—Cd1—N1—N2	106.4 (4)	Cd1—O1—C6—O2	38.1 (7)
O3^iii^—Cd1—N1—N2	−73.6 (4)	Cd1—O1—C6—C3	−140.8 (3)
O1—Cd1—N1—Cd2^vii^	−176.10 (19)	Cd2—O2—C6—O1	15.1 (7)
O1^i^—Cd1—N1—Cd2^vii^	3.90 (19)	Cd2—O2—C6—C3	−166.0 (3)
O3^ii^—Cd1—N1—Cd2^vii^	−87.11 (18)	C2—C3—C6—O1	9.4 (6)
O3^iii^—Cd1—N1—Cd2^vii^	92.89 (18)	C4—C3—C6—O1	−172.8 (4)
Cd1^vi^—O3—N4—C1	103.7 (4)	C2—C3—C6—O2	−169.6 (4)
Cd1^vi^—O3—N4—C5	−76.7 (4)	C4—C3—C6—O2	8.1 (6)

Symmetry codes: (i) −*x*, −*y*+1, −*z*+1; (ii) −*x*, −*y*+1, −*z*+2; (iii) *x*, *y*, *z*−1; (iv) −*x*, −*y*, −*z*+1; (v) *x*, *y*−1, *z*; (vi) *x*, *y*, *z*+1; (vii) *x*, *y*+1, *z*.

### Hydrogen-bond geometry (Å, °)

**Table:**

*D*—H···*A*	*D*—H	H···*A*	*D*···*A*	*D*—H···*A*
O4—H4C···N3^viii^	0.90	2.36	3.239 (7)	167
O4—H4B···O3^ix^	0.83	2.05	2.716 (4)	137

Symmetry codes: (viii) −*x*+1, −*y*+1, −*z*+1; (ix) −*x*+1, −*y*+1, −*z*+2.
